# Element-to-mineral-properties conversion (EMPC) via MARSpline for 3D geometallurgical modeling of the Koashva Apatite-Nepheline Deposit, Kola Peninsula, Russia

**DOI:** 10.1038/s41598-026-52558-9

**Published:** 2026-05-13

**Authors:** A. O. Kalashnikov, A. S. Ganyushkina, G. O. Nagovitsyn, N. G. Konopleva

**Affiliations:** 1 Murmansk Arctic University, Apatity Branch, 29, Lesnaya, 184209 Apatity Russia; 2 Geological Institute of the Kola Science Centre, Russian Academy of Sciences, 14, Fersman Street, Apatity, 184209 Russia; 3 FSBI All-Russian Scientific-Research Institute of Mineral Resources named after N M Fedorovsky (VIMS), Staromonetny per., 31, Moscow, 119017 Russia; 4 Mining Institute of the Kola Science Centre, Russian Academy of Sciences, 24, Fersman Street, Apatity, 184209 Russia

**Keywords:** Mineral deposit modelling, Machine learning, Geometallurgy, Kola Alkaline Province, Khibiny, Apatite, Environmental sciences, Solid Earth sciences

## Abstract

Efficient processing of complex ores is often hampered by an incomplete understanding of the spatial distribution of critical mineralogical properties. Here, we introduce a machine learning-based framework that addresses this gap. Using the multivariate adaptive regression splines (MARSpline) method, we develop predictive models for converting standard geochemical assays into mineralogical properties – a process we term Element-to-Mineral-Properties Conversion (EMPC). Applied to the Koashva apatite-nepheline deposit (Kola Peninsula, Russia), our approach yields reliable predictions (r typically > 0.85 for major minerals and > 0.6 for key trace elements in apatite) for mineral composition and ore mineral chemistry using only bulk-rock P_2_O_5_, Al_2_O_3_, and TiO_2_. These predictions enable the construction of the first 3D block models of mineral composition and mineral chemistry for this deposit. By integrating these models with conditional geometallurgical criteria, we identify and spatially delineate zones of refractory ore (containing > 6 vol% pyroxenes and > 1 vol% “liebenerite”) and areas yielding low-grade apatite concentrate (where apatite contains > 6.5 wt% SrO + REE_2_O_3_). This work establishes a generalizable, data-driven pathway for geometallurgical modeling, transforming historical exploration data into a critical decision-making tool for optimizing mine planning and mitigating processing challenges.

**Supplementary Information:**

The online version contains supplementary material available at 10.1038/s41598-026-52558-9.

## Introduction

The behavior of the ore being processed depends on various factors, including the mineral composition, rock texture, mineral chemistry, hardness of the rocks, rock fragmentation characteristics concerning size distribution and shape. Mineral properties and their relations is a key factor to control the ore quality within the technological processes^1–4^. This information is required both for ore body knowledge in situ (for planning a mining, mineral processing, output etc.) and for ore flow control (real-time ore quality control). The first is currently conceptualized as a geometallurgical approach. Geometallurgy integrates geological and mineralogical data with mineral processing parameters to predict ore behavior and optimize mine planning^5–10^.

Currently, there are three strategies of geometallurgical investigations of ore deposits, namely, (1) strategy based on interpolation of the technological sampling results, (2) proxy approach, (3) spatial-mineralogical approach^11–16^, and their combinations^14^.

1) The interpolation of technological properties determined from laboratory/low-volume samples. This approach is best-documented in^3,10,13,17–20^. This approach is useful in case of low variability of the technological properties of ores, as well as for operational planning during additional exploration of individual ore bodies and productive blocks of more complex deposits. However, this approach is also quite efficient for geometallurgical modeling of the entire deposit, when a single technological parameter is important, e.g., grindability.

2) The proxy approach. Technological parameters are calculated according to the dependencies of ore processing indicators on geological factors. This approach is described e.g. in works^11,19,21–27^. The task of calculating the mineral abundances (vol% or wt%) from whole-rock geochemistry is a part of the proxy-strategy, and is known as an Element-to-mineral conversion (EMC), see e.g.^28–31^.

3) The spatial-mineralogical strategy. It is based on the idea that the technological parameters of ore depend on the properties of the minerals comprising the ore (their content in rocks, chemical composition of the minerals, grain size, and dispersion of these features) and their relationship (see e.g. ^2,6,32–35^). Currently it is considered to be the most progressive research strategy^12,35^, yet its pure implementation is costly in terms of both time and money^31^. Moreover, the “calculation” of ore processing parameters from the mineralogical properties of a particular ore is a non-trivial problem that currently does not have a general solution.

Frenzel and colleagues^27^ showed that mineral chemistry of sulfide minerals can be predicted by the whole-rock chemistry. Recently, some researchers^23^ set a similar problem of predicting the ore dressing properties (yield of magnetite as a target mineral applying different methods of magnetic separation and different grinding parameters) by bulk-rock chemistry and suggested an approach to solve the problem using the machine learning methods.

Element-to-mineral conversion (EMC) refers to the estimation of mineral abundances (vol% or wt%) from whole-rock geochemistry. In contrast, we define Element-to-Mineral-Properties Conversion (EMPC) as the prediction of mineral-specific properties (such as the chemical composition of individual minerals like SrO in apatite, grain size, or mineral associations) from bulk-rock chemistry. While EMC targets how much of a mineral is present, EMPC targets what the mineral is like in terms of its composition and texture^36^. Previously, we studied the use of four regression methods (linear multiple regression, random forests, artificial neural networks, multivariate adaptive regression splines) to predict the chemical composition and average grain size of ore minerals (apatite, magnetite, baddeleyite) of the Kovdor Zr-P-Fe deposit (Murmansk Region, Russia), as well as the probability of their intergrowth with other minerals (the presence of inclusions of ilmenite and spinel in magnetite, the intergrowth of baddeleyite with pyrochlores)^36^. The multivariate adaptive regression splines (MARS) method turned out to be the best one. Moreover, the MARS method has an advantage of not imposing any requirements on any type or class of connections (for example, linear, etc.) between predictors and dependent variables. Moreover, while other methods like random forests or artificial neural networks can also capture non-linear relationships, they operate as “black boxes” and do not directly provide an interpretable regression function. MARSpline offers a compromise between flexibility and transparency, which is essential for geometallurgical applications where users need to understand why a certain prediction is made (e.g., which geochemical threshold triggers a change in apatite chemistry).

Therefore, in this work we implement and validate the MARSpline-based EMPC framework to construct the first high-resolution, mineralogy-based 3D geometallurgical model of the Koashva apatite-nepheline deposit (the Khibiny peralkaline massif, North-Western Russia, see details in section *Study area*,* materials and methods*). We demonstrate that this approach not only provides accurate predictions of mineral composition and chemistry but also enables direct spatial identification of refractory ore zones and areas yielding low-quality concentrate, thereby bridging the critical gap between traditional exploration data and operational metallurgical planning.

### Problem statement

Until recently, apatite-nepheline ores of the Khibiny massif were characterized by favourable processability, a high level of apatite extraction (93–95%), and high quality of the resulting commercial concentrate (> 39.5% P_2_O_5_). But in recent years the processing of apatite ores from the Khibiny deposits is disrupted from time to time by the occurrence of refractory ores. This is explained by the fact that every year the mining process involves deeper and deeper horizons of the deposits as well as relatively poor ores with a slightly different mineral composition and different relationship between minerals in ores than those previously involved in operation. Moreover, when processing ore from the zones of secondary alteration (e.g. so called spreustein zones – ijolite with altered nepheline and micas, zeolitization zones, etc.), it is very difficult to obtain high-quality apatite concentrate using flotation modes of the Apatite-Nepheline Ore Dressing Plants.

In general, several groups of factors can be distinguished that affect the Khibiny apatite-nepheline ore dressing.

1. Mineral composition of ores:

- reduced total content of the main ore minerals, i.e., apatite and nepheline;
- increasing content of the dark-colored minerals, i.e., clinopyroxenes, micas, lamprophyllites;
- the presence of clay and secondary minerals in the zones of secondary mineralization, e.g. “liebenerite” – mica pseudomorphous after nepheline.

2. Properties and composition of apatite:

- increased contents of isomorphic impurities (SrO, REE_2_O_3_, SiO_2_) and accordingly reduced contents of the main components (CaO, P_2_O_5_) in apatite from poor ores;
- grain size (presence of finely dispersed apatite less than 10 microns in size);
- the presence of close bimineral and polymineral intergrowths of apatite with nepheline, aegirine, feldspar, annite, lamprophyllite, and titanomagnetite; covering of apatite grains with the annite, clay minerals and iron hydroxides changes the surface properties of apatite.

3. Modern mineral formation after ore extraction. There is a steady tendency of increasing of the flotation pH up to 10.0-10.5. The most probable reason for this is an ingress of soluble sodium carbonates into the pulp, said carbonates being formed during the ore mining at the internal tectonic stress discharge zones37–40.

The problem of evaluation of the mineral composition of ores and the chemical composition of apatite using regression equations from the components of ordinary sampling was first set and partially solved for the Khibiny deposits back in 1980s. This was facilitated by a set of components of an ordinary sampling from exploratory boreholes, which included P_2_O_5_, Al_2_O_3total_, Al_2_O_3acid−soluble_ (aluminum contained in acid-soluble feldspathoids and zeolites), and TiO_2_. This set of components makes it possible to estimate the content of the main minerals in the rocks of the deposit.

From 97 to 100 wt% of P_2_O_5_ that occurs in various types of ores and host rocks of the Koashva deposit is concentrated in apatite. Therefore, the content of apatite is related by direct functional dependence to the content of P_2_O_5_ (C_Аp_ = 2.451 × C_P2O5_). Nepheline and feldspar are the main minerals-concentrators of aluminum in the productive zone of the deposit. Nepheline accounts for 87–97 wt% Al_2_O_3total_ and 90–100 wt% Al_2_O_3acid−soluble_. This makes it possible to estimate the nepheline content in rocks by the content of Al_2_O_3acid−soluble_. Accordingly, the difference between Al_2_O_3total_ and Al_2_O_3acid−soluble_ (ΔAl_2_O_3_) characterizes the feldspar content in rocks with a sufficiently high reliability. Titanium dioxide is concentrated mainly in titanite (59–86%) and titanomagnetite (4–30%)^41^. This methodology for assessing the contents of minerals and useful components by correlation with the basic characteristics (Р_2_О_5_ and Al_2_O_3 total_) has been introduced into the practice of calculating the reserves of the Khibiny deposits.

A problem of predicting the properties of minerals by the chemical composition of rocks was first set and partially solved for in 1980s on the example of the Khibiny titanite-apatite-nepheline deposits^41,42^. However, computational power was poorly developed at that time and progressive methods for regression modeling were at an early stage of development.

The core of the problem, therefore, is not a lack of understanding, but a fundamental data translation gap. We possess vast archives of historical geochemical data (P₂O₅, Al₂O₃, TiO₂), but we lack the means to convert them efficiently and accurately into the 3D mineralogical models required for predictive geometallurgy. The classical approaches, reliant on linear assumptions, are inadequate for capturing the mineralogical complexity of the ore body. This work closes this gap by demonstrating how the Element-to-Mineral-Properties Conversion (EMPC), powered by machine learning, can transform legacy data into a dynamic, three-dimensional geometallurgical decision-making tool.

## Results

### Input data

To predict the mineral composition of rocks and the mineral chemistry, data from an ordinary core sampling of exploration boreholes were used, namely, P_2_O_5_, Al_2_O_3_, Al_2_O_3a.s_. (acid soluble), and TiO_2_. Descriptive statistics of training and predicting set are shown in Table 1.

**Table 1:** Mineral composition of rocks, rock chemistry and mineral chemistry of the training set (a), rock chemistry of the predicting set (b).

Component (*N* of analyses)	Min	Lower quartile	Median	Mean	Upper quartile	Max	Coef. variation
(a) Mineral and chemical composition of rocks, the training set							
Minerals, wt%							
Nepheline (824)	1.82	29.12	44.19	42.30	55.65	84.36	41.9
Pyroxenes (824)	0.34	5.56	10.69	12.13	17.09	56.79	67.2
Titanite (824)	0.07	2.18	4.51	7.12	8.32	61.44	117.6
Titanomagnetite (824)	0.00	0.40	1.10	2.69	3.20	66.00	169.1
Potassic feldspar (824)	0.01	0.56	2.05	4.64	5.33	42.81	146.5
Ilmenite (824)	0.000	0.005	0.008	0.211	0.240	3.130	195.7
Annite–phlogopite (824)	0.000	0.020	0.260	0.658	0.880	10.770	160.8
Amphiboles (824)	0.003	0.003	0.059	0.170	0.099	7.350	297.2
Lamprophyllite (824)	0.000	0.000	0.000	0.067	0.002	3.260	329.9
“Libenerite” (824)	0.003	0.003	0.003	0.394	0.003	23.100	579.8
Rock chemistry, wt%							
P_2_O_5_ (824)	0.18	2.36	8.43	11.86	19.72	36.69	87.3
Al_2_O_3_ (824)	2.04	9.83	15.28	14.75	19.80	28.76	41.3
Al_2_O_3a.s_. (824)	0.73	8.93	14.13	13.36	17.86	27.77	43.6
TiO_2_ (590)	0.31	1.62	2.65	3.89	5.05	26.8	92.8
Apatite chemistry, wt%							
SrO (241)	2.00	3.25	4.30	4.16	5.00	6.40	27.4
F (237)	2.85	3.07	3.18	3.18	3.28	3.48	3.9
REE_2_O_3_ (237)	0.60	0.93	1.05	1.05	1.15	1.70	16.4
Nepheline chemistry, wt%							
Al_2_O_3_ (42)	31.32	32.05	32.34	32.33	32.68	33.17	1.4
Ga_2_O_3_ (91)	0.0035	0.005	0.0057	0.0060	0.0068	0.0110	21.6
Rb_2_O (84)	0.008	0.012	0.014	0.017	0.017	0.062	57.5
Titanite chemistry, wt%							
Nb_2_O_5_ (79)	0.121	0.298	0.320	0.323	0.340	0.495	14.8
Titanomagnetite chemistry, wt%							
Fe_total_ (61)	53.03	57.77	58.61	58.41	59.72	60.84	2.5
(b) Rock chemistry, predicting set							
P_2_O_5_ (51268)	0.01	1.33	6.20	10.34	17.88	41.88	100.4
Al_2_O_3_ (40134)	0.16	12.28	17.07	16.17	20.86	38.75	35.6
Al_2_O_3a.s_. (29496)	0.01	11.35	15.47	14.83	19.00	31.58	36.9
TiO_2_ (12233)	0.08	1.66	2.43	2.81	3.32	21.10	71.1

Al_2_O_3a.s_. – Al_2_O_3_ acid-soluble.

Since the different quantities of components were analyzed in different samples of the predicting set, we had to find respective regression models for each set of components. In total there were six variants of sets of predictors (chemical components) that occurred in the data of ordinary sampling:

- (1s) P_2_O_5_, Al_2_O_3tot_, Al_2_O_3a.s_., TiO_2_ (12064 samples of ordinary sampling).
- (2s) P_2_O_5_, Al_2_O_3tot_, Al_2_O_3a.s_. (17426 samples)
- (3s) P_2_O_5_, Al_2_O_3tot_ (10608 samples).
- (4s) P_2_O_5_, Al_2_O_3tot_, TiO_2_ (33 samples).
- (5s) P_2_O_5_, TiO_2_ (135 samples).
- (6s) P_2_O_5_ (10999 samples).

### Predicting of the mineralogical properties

A regression model was found for each variable and for each set of predictors. In total, 108 regression models were built, the models (MARSpline equations) are presented in Supplementary Materials 1. The quality of prediction of the desired ore properties (i.e., correlation coefficients) for different sets of predictors is shown in Table 2 for minerals in the rock and in Table 3 for chemical compounds in the mineral. Other regression error metrics (r^2^, MAE and RMSE, for the predictor set 1) are presented in subsection Model Validation.

The p-value of 6 regression models turned out to be more than 0.05, thus they were rejected.

**Table 2:** Correlation coefficients of actual and predicted mineral contents in the rock for all predictor sets (1–6 s).

Predictor set	Nepheline	Pyroxenes	Titanite	Magnetite	Feldspar	Ilmenite	Mica	Amphibole	Lamprophyllite	«Liebenerite»
1s	0.99	0.92	0.98	0.85	0.85	0.45	0.39	0.32	0.35	0.79
2s	0.99	0.87	0.87	0.69	0.88	0.36	0.37	0.34	0.41	0.75
3s	0.97	0.85	0.86	0.67	0.64	0.36	0.3	0.27	0.33	0.26
4s	0.97	0.91	0.98	0.85	0.7	0.43	0.34	0.3	0.32	0.21
5s	0.92	0.78	0.97	0.63	0.61	0.39	0.3	0.26	0.26	0.21
6s	0.81	0.70	0.51	0.30	0.53	0.26	0.15	0.20	0.20	0.21
Ivanov 1987	0.88–0.996	0.62–0.93	0.78–0.98	0.39–0.85	0.29–0.90					

The green color highlights the best predicted minerals (*r* ≥ 0.6). Yellow means *p* < 0.05 and *r* < 0.6. Dashes mean that the significance level is *p* > 0.05 (red). Ivanov 1987 stands for multiple correlation coefficients from the first set of predictors calculated for different types of ores and rocks in^41^.

Based on the data in the table, the following observations can be made:

(1) Nepheline and pyroxene contents are the most predictable ones (in all six sets of predictors);

(2) For titanite, magnetite, feldspar (from the 1s to 5s sets of predictors), and “libenerite” (in the 1s and 2s sets) the value of the correlation coefficients r is also more than 0.6;

(3) The correlation coefficients of the measured and calculated contents for other minerals are smaller, but they are significant, i.e. p < 0.05.

**Table 3:** Correlation coefficients of actual and predicted contents of chemical compounds in minerals for all predictor sets (1–6 s).

	Apatite			Nepheline			Titanite	Magnetite
	SrO	REE_2_O_3_	F	Ga_2_O_3_	Rb_2_O	Al_2_O_3_	Nb_2_O_5_	Fe_total_
1s	**0.87**	**0.76**	*0.37*	**0.81**	**0.78**	**0.60**	**0.63**	**0.76**
2s	**0.84**	**0.70**	*0.37*	*0.46*	**0.68**	**0.60**	**0.63**	-
3s	**0.83**	**0.68**	*0.3*	*0.44*	*0.23*	**0.60**	*0.36*	-
4s	**0.85**	**0.76**	*0.34*	*0.40*	*0.23*	*0.35*	*0.36*	**0.64**
5s	**0.85**	**0.62**	*0.30*	*0.40*	-	*0.35*	*0.43*	**0.64**
6s	**0.72**	*0.48*	*0.30*	-	-	*0.35*	*0.36*	-
Perekrest 1985	0.69	0.47	0.30					

The Bold highlights the most correlated components with values of *r* ≥ 0.6. Italic means *p* < 0.05 and *r* < 0.6. Dashes mean that the significance level is *p* > 0.05. Perekrest 1985 stands for the correlation coefficients of components from the sixth set of predictors (P_2_O_5_) calculated using a quadratic regression model in ^42^.

Analyzing the table data, we can say that:

(1) The contents of associated components in apatite are predicted with varying but sufficient accuracy for any set of independent variables. The SrO content in apatite will be well predicted in each set of predictors, while the REE2O3 content will be well predicted by the 1–5 s set, and slightly worse by the 6s set. The main predictor for apatite chemistry is P2O5 content in a rock.

(2) The content of Ga2O3 in nepheline is the best predicted by the 1s set of predictors, and the content of Rb2O in nepheline may be most accurately predicted by the 1st and 2nd sets of predictors. The main predictor for nepheline chemistry is Al2O3 tot. and Al2O3 a.s. content in a rock.

(3) The content of Nb2O5 in titanite will be predicted according to the 1s and 2s sets of predictors. The remaining sets of predictors show smaller, but still significant, correlations.

(4) The content of Fe in titanomagnetite will be predicted by the 1s, 4s, and 5s sets of predictors. The main predictor for apatite chemistry is TiO2 content in a rock. This is explained by the fact that magnetite contains a significant amount of titanium, which isomorphically replaces iron.

As expected, the prediction quality generally improves with the number of predictors (Tables 4 and 5). The full set of predictors (1s: P_2_O_5_, Al_2_O_3tot_, Al_2_O_3a.s_., TiO_2_) yields the highest r for most targets. However, even with a single predictor (6s: only P_2_O_5_), the r for nepheline, pyroxenes, and SrO in apatite remains above 0.81, 0.70, and 0.72 respectively (Tables 2 and 3), demonstrating that useful predictions can be made from incomplete historical data.

### Real data vs. regression models

To compare the mineral composition of rocks by regression models and direct analyses, a control sample of 18 samples of rocks and ores from the Koashva deposit was used. The control sample set contains direct determinations of chemical and mineral composition. The 2s predictor set (P_2_O_5_, Al_2_O_3 tot_. and Al_2_O_3 a.s_) was used in the regression models.

For each sample, we computed sample-level prediction (the mineral composition predicted by the regression model using the sample’s own rock chemistry, P_2_O_5_, Al_2_O_3tot_, Al_2_O_3a.s_.), and sample-level measured (the mineral composition determined by direct phase analysis on the same sample).

**Fig. 1:**
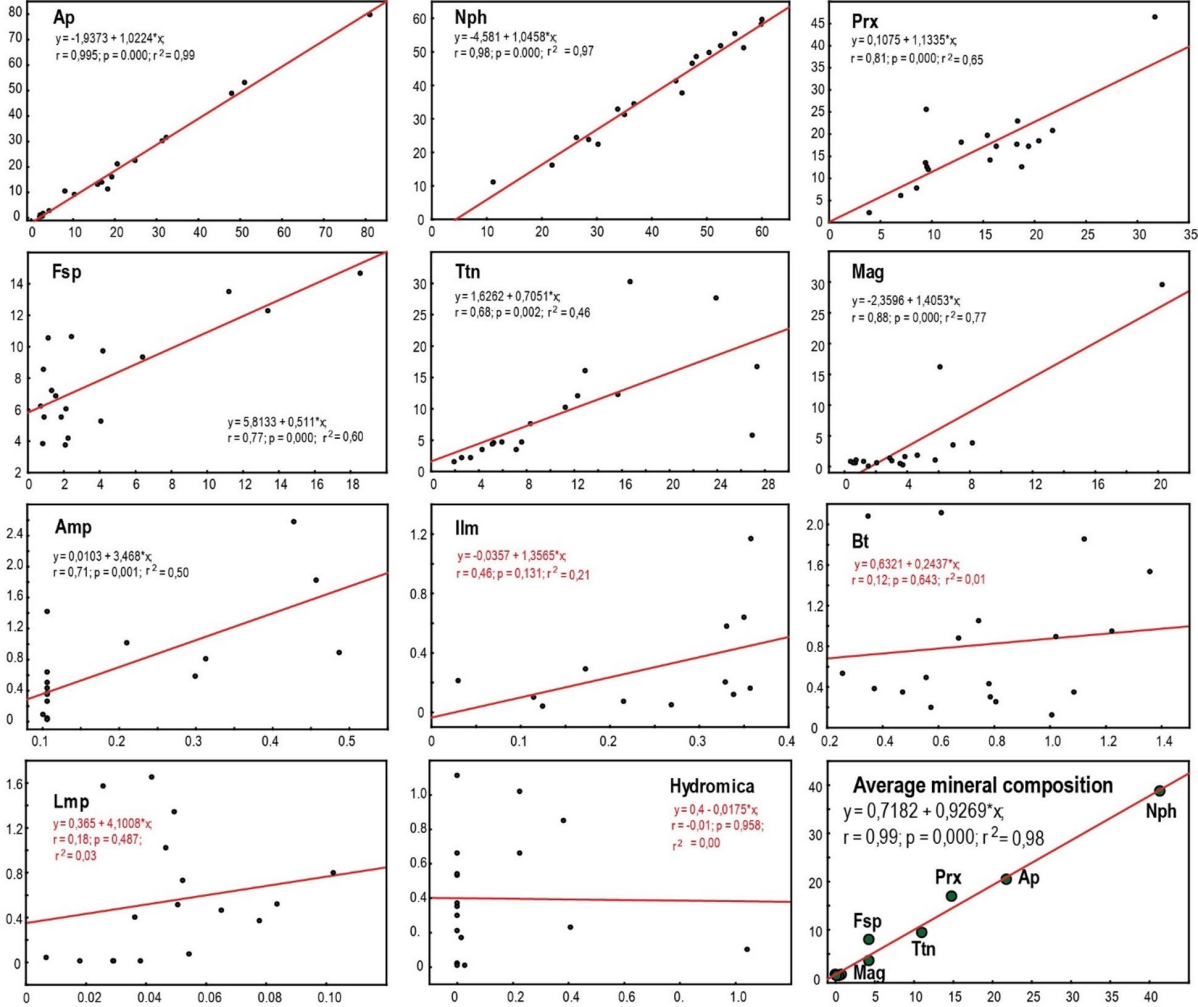
Comparison of the mineral composition of rocks: direct measurements (Y-axis) vs. regression models data (X-axis).

To assess block-scale performance, we then calculated the arithmetic mean of these 18 sample-level values (both predicted and measured) and compared the two means (shown in Fig. 1, the last panel). The high correlation (*r* = 0.99) refers to this comparison of averages, not to the sample-by-sample correspondence.

Due to the small size of the control set (*n* = 18), we did not compute sample-level RMSE. The primary purpose of this control comparison was to verify the absence of systematic bias at the block scale (averaged values). For a more rigorous sample-level evaluation, a larger independent test set would be required, which is a direction for future work.

In geometallurgical modeling, the primary unit of decision-making is the mining block (typically thousands to hundreds of thousands of tonnes), not an individual core sample. The block-scale mineral composition is estimated by averaging predictions over many samples within the block. Therefore, the relevant performance metric is the accuracy of the mean estimate, not the error on a single sample. The high correlation (*r* = 0.99) between predicted and measured average compositions confirms that the models are suitable for block-scale estimation. Sample-level errors are smoothed by the subsequent kriging interpolation and block averaging.

### Mineralogical and geometallurgical block modeling

The predicting and modeling of the distribution of mineralogical properties in the volume of the Koashva deposit were carried out in several steps.

**Fig. 2:**
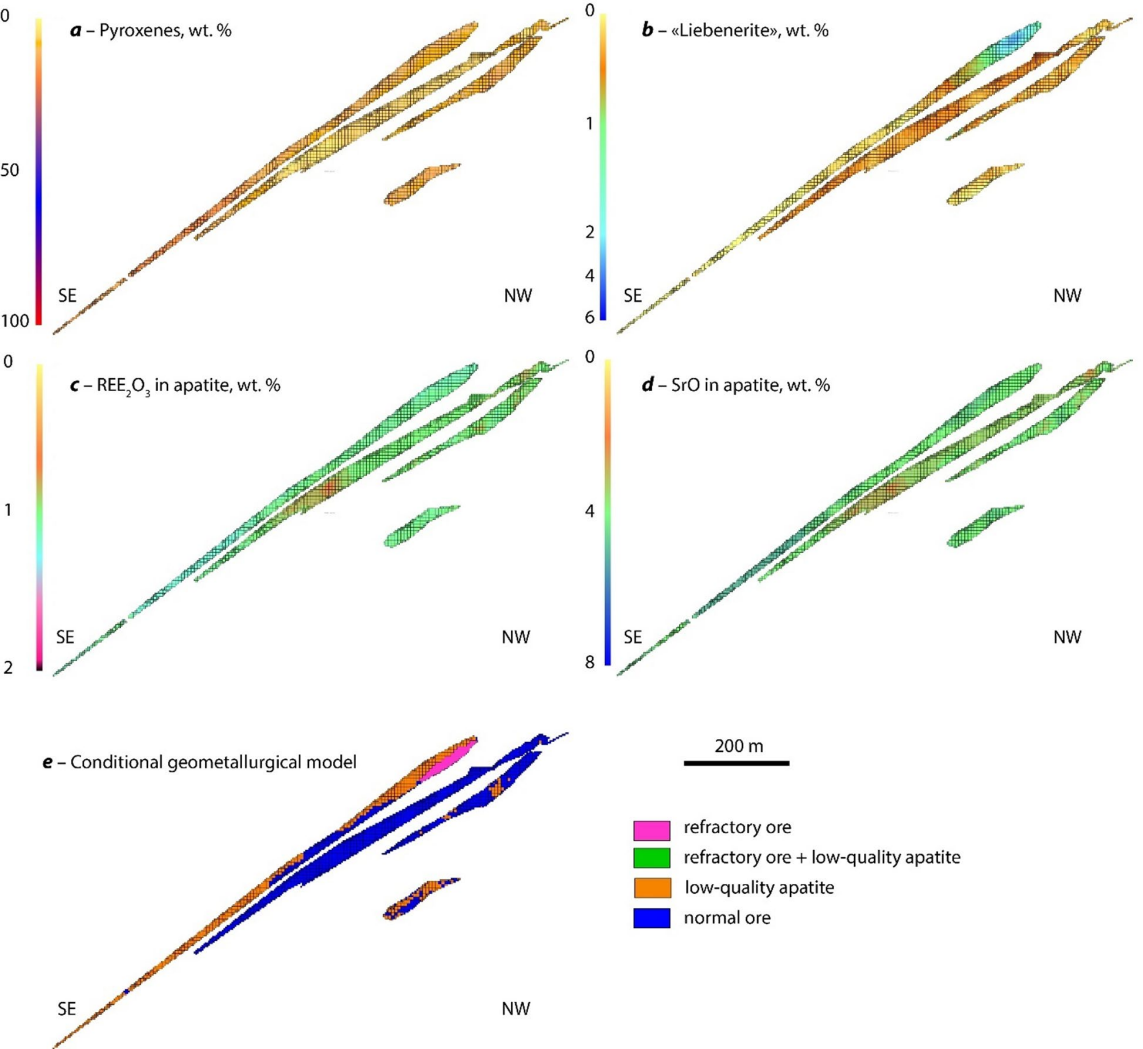
Cross-sections of interpolation block models of the predicted mineralogical properties: (**a**) content of pyroxenes, (**b**) content of “liebenerite”, (**c**) REE_2_O_3_ in apatite, (**d**) SrO in apatite; all in wt%, (**e**) conceptual geometallurgical model of the Koashva deposit. Predictions used the maximum available predictor set (1–6 s) for each sample location (see Results – Input Data).

1. Data from the ordinary sampling were divided into groups according to a set of chemical components (predictors) as specified in subsection “Input data”. Each group had its respective regression model.

2. The sampling results were divided into composite intervals with an average length of 3 m.

3. By means of variography, the interpolation parameters were found for each of the predicted mineralogical properties according to the composite intervals. An interpolation block model was built pursuant to these parameters. Cross-sections of some mineralogical variables (content of pyroxenes and “liebenerite”; REE2O3 and SrO content in apatite) are shown in Fig. 2a-d.

In our case a refractory ore can be defined as follows: the content of “liebenerite” is more than 1 vol% and the content of aegirine is more than 6 vol%. It is also possible to separate ore with low-quality apatite that in any case will be processed into a low-grade concentrate, i.e., the content of SrO + REE_2_O_3_ in apatite is more than 6.5 wt%. The thresholds are derived from previous technological studies on Khibiny ores^38–40,42,43^. Specifically, pyroxenes threshold corresponds to increased flotation reagent consumption and reduced apatite recovery. “Liebenerite” threshold corresponds to the level where secondary alteration begins to significantly depress apatite flotation. SrO and REE_2_O_3_ in apatite threshold empirically linked to a decrease in apatite concentrate grade below standard P_2_O_5_ level. These thresholds are site-specific to the Koashva deposit and the current processing flowsheet. They are used here to demonstrate the capability of the EMPC workflow to generate spatially explicit geometallurgical classifications. For operational use, these thresholds would require re-evaluation and calibration against laboratory tests and plant performance data.

A formal sensitivity analysis of the classification thresholds is beyond the scope of this revision, as it would require expanded extractive metallurgical tests. We acknowledge this as a limitation of this work. In practice, prior to applying the geometallurgical model for mine planning, the thresholds should be calibrated using a designed set of flotation tests on representative samples, and a sensitivity analysis should be performed to quantify the impact on geometallurgical classification of ores. This is a direction for future work.

The separation of these ore types is performed by logical calculations in accordance with the specified boundary conditions. If these conditions are combined, it is possible to construct a geometallurgical model of the deposit with indications of refractory ore zones and zones of low-quality concentrate output (Fig. 2e). Similarly, by adding the necessary boundary conditions it is possible to carry out more detailed typification of ores in the volume of the deposit in accordance with the current or potential requirements of processing technologies and/or production economics. The final integrated geometallurgical model (Fig. 2e) thus provides a direct, spatially explicit tool for mitigating the processing challenges outlined at the beginning of this study.

## Discussion

Our study demonstrates that the MARSpline-based EMPC approach successfully transforms standard geochemical data into a high-resolution, predictive model of mineralogical properties, enabling the construction of a practical 3D geometallurgical model for the Koashva deposit. This work bridges the long-standing gap between readily available geochemical assays and the mineralogical data essential for forecasting processing performance.

The good performance of the MARSpline method for this task likely reflects its ability to capture complex, non-linear relationships between rock chemistry and mineralogical properties. For the Koashva dataset, MARSpline outperformed linear and quadratic regression approaches^41,44^. However, this conclusion is specific to the current dataset; direct comparisons on other deposits would be needed to claim general superiority. Furthermore, unlike “black box” models such as neural networks, the MARSpline models remain interpretable, providing insight into the key geochemical thresholds that control mineral distribution and chemistry. This balance of high accuracy and interpretability makes it particularly suitable for geometallurgical applications where model transparency is crucial for stakeholder trust. This approach has the potential to facilitate geometallurgical re-evaluation of deposits with extensive historical geochemical databases, potentially reducing the need for new, costly mineralogical sampling campaigns. Nevertheless, successful application to other deposits would require careful validation and, ideally, retraining of models with local data. By converting historical data into a mineralogical context, our approach unlocks a vast, untapped resource for strategic mine planning. Note that the chosen thresholds of mineral content and mineral chemistry are not intended to be transferable to other deposits or even to different processing flowsheets at the same deposit. Mineralogical controls on flotation behavior vary with ore type, reagent regime, and grinding characteristics. Therefore, the numerical values should not be used outside the context of the Koashva apatite-nepheline ore processing as practiced today. However, the approach (defining logical rules based on mineralogy and rock chemistry) is fully transferable. For a different deposit, one would determine its own thresholds through targeted metallurgical testing.

The resulting geometallurgical model (Fig. 2e) moves beyond a static geological description to become a dynamic decision-making tool. The identification of refractory ore zones and areas with low-quality apatite is not merely an academic exercise; it provides a direct, spatially explicit input for mitigating operational risks. For instance, knowing the location and volume of problematic ore allows for proactive blending strategies or the scheduling of selective mining and processing. Crucially, the model’s ore type classification is based on fundamental mineralogical criteria. Consequently, as processing technology evolves or economic conditions change (i.e., if it will be possible to implement potential ore processing flows indicated in Fig. 5 by red lines), the model can be rapidly updated by simply adjusting the classification rules, ensuring its long-term relevance without the need for re-drilling or re-assaying.

Looking forward, several avenues for further development emerge. A key to further enhancing the model’s accuracy lies in the input data. The absence of data for all four components (P_2_O_5_, Al_2_O_3total_, Al_2_O_3a.s_., TiO_2_) in core samples reduces the prediction accuracy. To improve accuracy, it is necessary to use analytical methods that allow the determination of a bigger number of components in ordinary sampling, for example, XRF, ICP AES/MS etc. For example, in earlier work^36^ we showed that for sufficiently high accuracy of prediction of mineralogical properties of the Kovdor baddeleyite-apatite-magnetite deposit ores it is necessary to determine 7–10 rock-forming chemical components.

Consequently, the current model’s accuracy is contingent on the representativeness of the training set; expanding this dataset to cover a wider range of ore types and alteration styles would enhance its robustness. The logical next step is the integration of these predictive mineralogical models with actual processing performance data from the plant, creating a true quantitative link from geology to metal recovery. Finally, while validated here for a specific apatite-nepheline deposit, the EMPC framework is inherently generalizable. Its application to other complex, multi-commodity deposits represents a promising frontier for maximizing resource efficiency and operational resilience across the mining industry.

The performance of the EMPC models depends critically on the representativeness of the training data. Our training set (Table 1a) covers the main ore types of the Koashva deposit but does not include all possible alteration varieties (e.g., intensely zeolitized zones). Predictions for samples that fall outside the range of predictor values present in the training set (e.g., TiO₂ > 27 wt%) may be unreliable. Additionally, the models are specific to the analytical protocols used for the training data (titrimetry for Al_2_O_3_, photocolorimetry for TiO_2_, etc.). If analytical methods change (e.g., from titrimetry to ICP AES), systematic biases may appear and require recalibration. Finally, the geological and mineralogical setting of the Koashva deposit (alkaline apatite-nepheline ores) is unique. The models cannot be assumed to work for other deposit types (e.g., sedimentary phosphorites or carbonatite-phoscorite Fe-P deposits) without retraining.

Note that our models have significant limitations in predicting minor minerals. As shown in Tables 2 and 5, prediction accuracy for minor minerals (mica, amphibole, lamprophyllite, ilmenite) is low (*r* < 0.4). This is expected for several reasons. First, these minerals occur in low abundances (median < 1 wt%, Table 1a), and their analytical determination in the training set has higher relative uncertainty. Second, their geochemical signatures are subtle and may be masked by major minerals (e.g., TiO_2_ is partitioned among titanite, titanomagnetite, and lamprophyllite). Third, the predictor set with four components maximally may not contain sufficient information to resolve these minor phases independently. While these minor minerals are not the primary drivers of refractory behavior or concentrate grade at the Koashva deposit, their low prediction accuracy should be noted if future geometallurgical criteria include them. In the current model, they are not used for classification, so the main conclusions remain unaffected.

As for the presented geometallurgical model, we acknowledge that is entirely conditional, and the chosen their thresholds of key mineralogical parameters are illustrative. Numerical parameters values were not optimized, and a sensitivity analysis of the classification output was not performed. Prior to operational use, the thresholds should be calibrated using metallurgical test data, and the impact of prediction uncertainty on classification should be quantified.

The results presented here are strictly demonstrated for the Koashva deposit and the specific set of analytical methods used in exploration (titrimetry, photocolorimetry, etc.). The predictive models are not intended for direct transfer to other deposits without recalibration. However, the methodology (the EMPC workflow using MARSpline) is generalizable. For a new deposit, one would need to collect a training set of paired geochemical and mineralogical data, then re-train the models. Therefore, the main contribution of this study is the validation of a transferable workflow, not a set of universal regression equations.

## Conclusions

This study demonstrates that the integration of machine learning-based Element-to-Mineral-Properties Conversion (EMPC) with 3D geological modeling creates a powerful and generalizable framework for geometallurgy. By applying the MARSpline method to the Koashva deposit, we have successfully bridged the critical gap between historical geochemical data and the mineralogical properties that dictate processing performance.

Our key findings are as follows:

1. Reliable prediction from limited data for the Koashva deposit. The MARSpline method provides strong predictive performance (*r* > 0.9 for key minerals like nepheline, titanite and pyroxenes) using standard assay data (P_2_O_5_, Al_2_O_3_, TiO_2_). Prediction accuracy improves with the number of predictors, even with a single predictor (P_2_O_5_) useful models (*r* > 0.7 for nepheline, pyroxenes and SrO in apatite) can be obtained, suggesting applicability to some historical datasets, though performance will depend on the geological context and data quality.
2. A workflow for 3D mineralogical modeling. We demonstrate a complete workflow from geochemical data to 3D block models of mineralogical properties for the Koashva deposit, including the content of ore and gangue minerals as well as the chemical composition of apatite, nepheline, titanite, and titanomagnetite. This workflow extends traditional geological modeling and can be adapted to other deposits with appropriate training data.
3. From mineralogy to practical geometallurgy. The constructed 3D models enabled the development of a conceptual geometallurgical model for the Koashva deposit. By defining boundary conditions based on mineralogical criteria (e.g., > 6 vol% pyroxenes and > 1 vol% “liebenerite” for refractory ore; >6.5 wt% SrO+REE_2_O_3_ in apatite for low-grade concentrate), we spatially delineated zones of potential processing challenges, transforming abstract mineralogical data into a practical risk-mitigation tool.

The EMPC democratizes access to high-resolution mineralogical information by leveraging existing geochemical data, thus avoiding the high costs of extensive mineralogical sampling. This approach provides a dynamic and adaptable tool for strategic mine planning, ore blending, and processing optimization, ultimately enhancing the economic efficiency and sustainability of mining complex, multi-component ore deposits. The method’s generalizability promises wide applicability beyond apatite-nepheline ores to other deposit types.

## Study area, materials and methods

### Geological settings

**Fig. 3:**
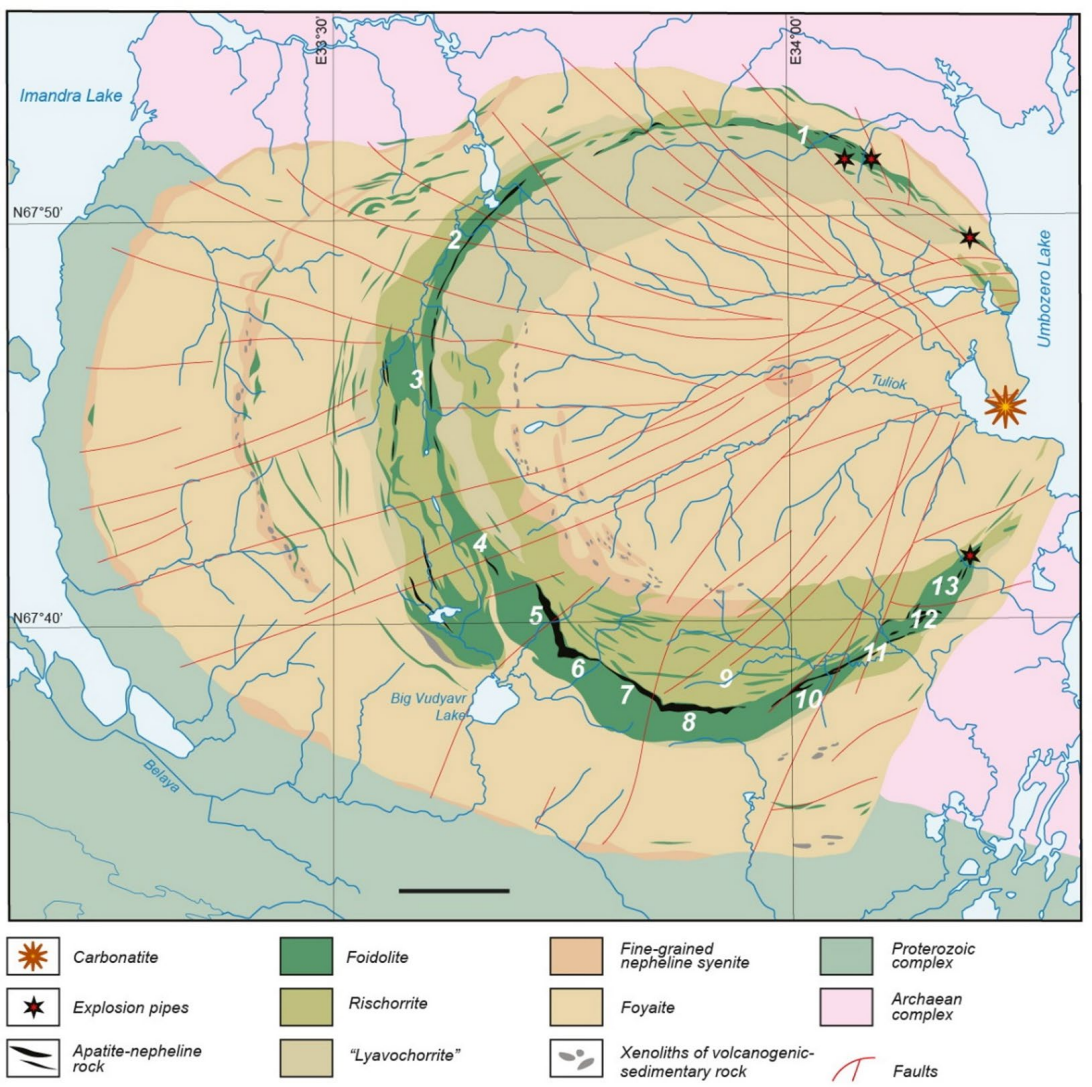
Simplified geological map of the Khibiny massif, modified after^45,46^. White numbers are apatite-nepheline deposits and occurrences: 1 – Valepakhk; 2 – Partomchorr; 3 – Kuelporr; 4 – Snezhny Tsyrk; 5 – Kukisvumchorr; 6 – Yuksporr; 7 – Apatitovy Tsyrk; 8 – Plato Rasvumchorr; 9 – Eveslogchorr; 10 – Koashva; 11 – Vuonnemyok; 12 – Nyorkpakhk; 13 – Oleny Ruchey.

The Khibiny alkaline massif is located in the western part of the Kola Peninsula at the contact of Proterozoic rocks of the Imandra-Varzuga greenstone belt with the Archean metamorphic complexes of the Kola-Norwegian megablock (Fig. 3).

The most common rocks of the Khibiny massif are nepheline syenites, i.e., foyaites. The foyaite body is divided into two approximately equal parts by the Main Ring zone of foidolites (melteigite-urtites). The foidolites are bordered by a rim of potassium-enriched nepheline syenites (rischorrite) and/or uneven-grained nepheline syenites (“lyavochorrite”). The apatite-nepheline and titanite-apatite-nepheline rocks form lens-like and stockwork deposits in the apical parts of the foidolite unit and are associated with it by gradual transitions.

The Khibiny apatite-nepheline deposits, which are the main source of phosphate raw materials in Russia, are of great importance for Russia, Europe, Latin America, India, and other regions^47,48^. The reserves of multicomponent apatite-nepheline ores of the Khibiny deposits comprises the main component (P_2_O_5_), associated minerals (nepheline, titanite and titanomagnetite) and elements in minerals (SrO, REE_2_O_3_, and F in apatite; Al_2_O_3_, Ga_2_O_3_, Rb_2_O in nepheline; TiO_2_ and Nb_2_O_5_ in titanite; Fe_total_ and TiO_2_ in titanomagnetite)^42^.

All the Khibiny apatite-nepheline deposits have a number of common geological, petrographic-mineralogical, and geochemical features, i.e., the similarity of geological position, unity of material composition and texture types of ores, composition of ore-forming minerals, etc. At the same time, they differ in features of localization and internal structure of the ore zone, morphology of deposits, and the degree of discontinuity of mineralization. Thus, three ore fields are distinguished within the productive ijolite-urtite arc, i.e., the South-Eastern, South-Western, and North-Western ones^49,50^. The deposits within each of these fields have similar structure.

**Fig. 4:**
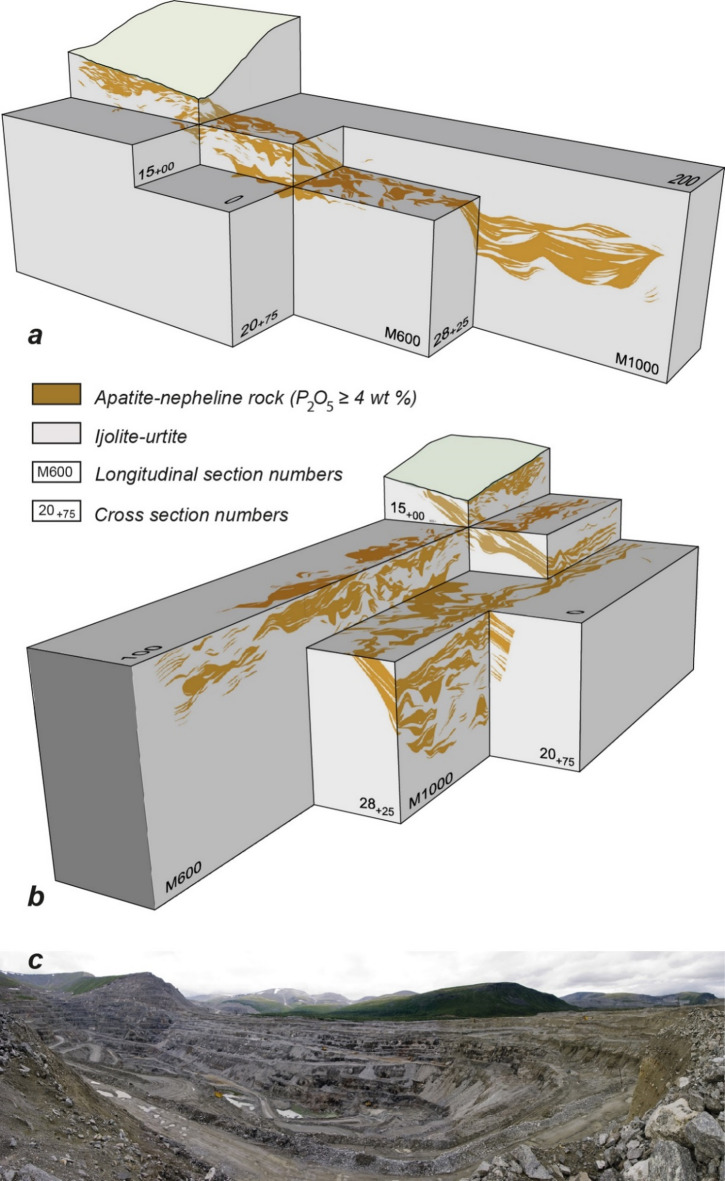
3D models of the Koashva deposit: (**a**) view from the south and (**b**) the east, modified after^51^; (**c**) a general view of the Koashva open pit (photo by Prof. Gregory Yu. Ivanyuk).

The largest deposit of the South-Eastern ore field is Koashva. The ore zone of the deposit is a fairly compact linear stockwork of lens-veined bodies of apatite-nepheline rocks (Fig. 4a, b) among orthoclase-bearing urtites (predominating) and ijolites^45,51,52^. The deposit is being developed by an open pit (Fig. 4c).

**Fig. 5:**
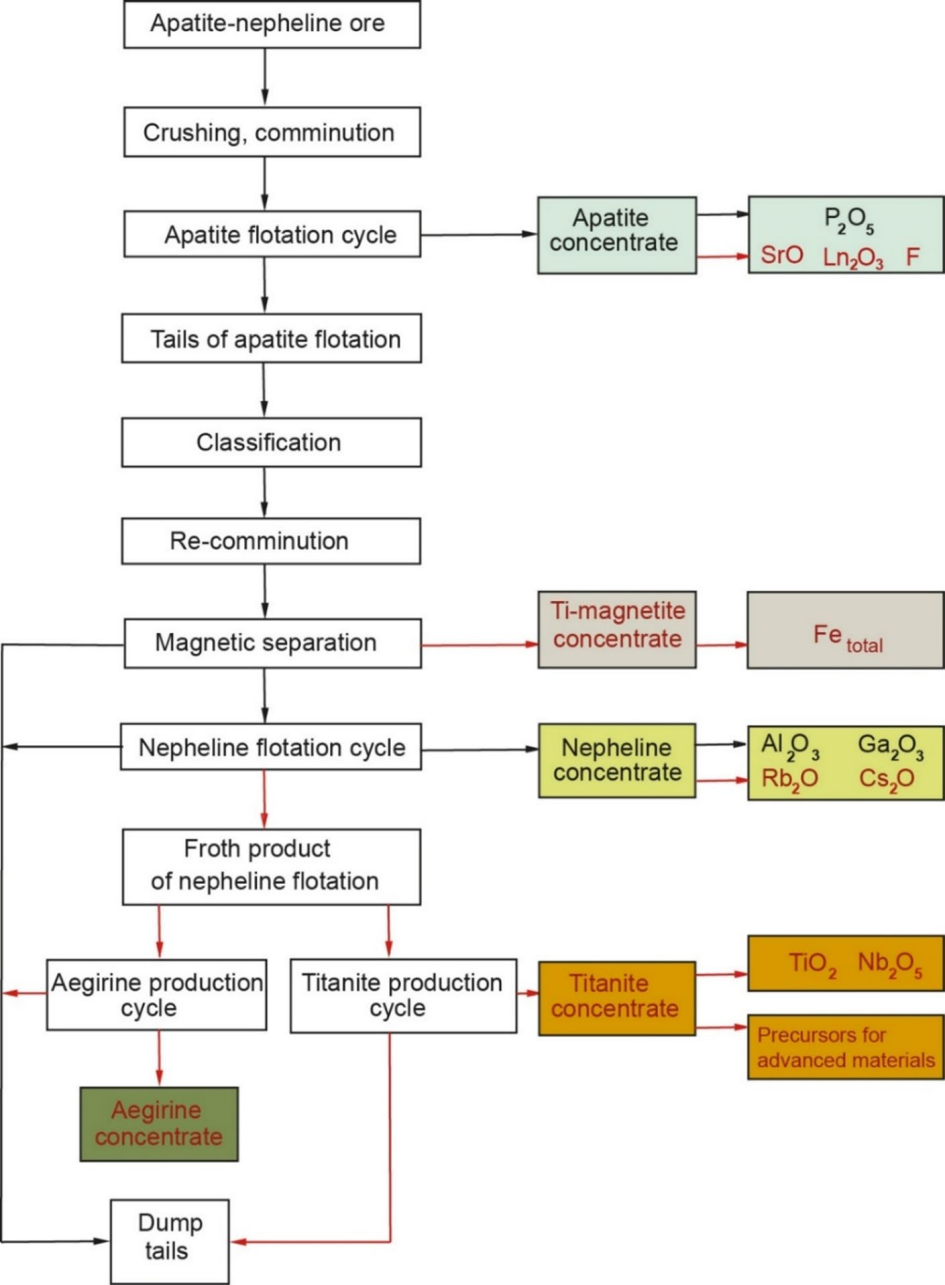
Flowchart of the Khibiny nepheline-apatite ore processing, synthesized after^42,43,53^. Red arrows show potential products of the ore processing. Colored rectangles are products.

The Khibiny ores are multicomponent, but currently only apatite (as a source of P_2_O_5_) and nepheline (as a source of Al_2_O_3_) concentrates are extracted from them. The rest of the useful components are sent to the tailings dump. The general scheme of the ore dressing process, including existing and potential processing lines, is shown in Fig. 5.

### Materials and analytical methods

The work is based on the data from detailed exploration of the deep horizons of the Koashva deposit^42^. All analyses were carried out in Murmanskaya GRE (Apatity, Russia), and external control was performed in Sevzapgeologiya (Saint-Petersburg, Russia). The results of phase mineralogical analysis (contents of nepheline, pyroxenes, titanite, magnetite, potassium feldspar, ilmenite, micas of the annite–phlogopite series, amphiboles, lamprophyllite, “liebenerite”) and chemical analyses of monofractions (apatite, nepheline, titanite and titanomagnetite) were used as a training set.

The contents of SrO, REE_2_O_3_, and F in apatite; Ga_2_O_3_, Rb_2_O, Al_2_O_3_ in nepheline; Nb_2_O_5_ in titanite; Fe_total_ in titanomagnetite were determined.

P_2_O_5_ was measured using titrimetry (for a concentration value of 1–30 wt%) and photocolorimetry (0.01–1 wt%). Al_2_O_3_ and Al_2_O_3a.s_. were measured by means of titrimetry, and TiO_2_ was measured by means of photocolorimetry. Fluorine in apatite was measured by an ion-selective electrode; strontium was measured by means of atomic absorption spectroscopy with atomic emission spectroscopy external control; the rare earths were measured by means of photocolorimetry. Gallium and rubidium in nepheline were analysed by the atomic emission spectroscopy method, and aluminum was analysed by the titrimetry method; niobium in titanite by the X-ray fluorescence method, and iron in magnetite was analysed by the titrimetry method.

### Data preprocessing

All geochemical and mineralogical data were used in original units (wt%) without scaling or transformation. Samples with missing values for any predictor in a given predictor set (1–6 s) were excluded from training for that set. No outlier removal was performed to preserve natural variability. For the predicting set (large exploration database), samples with incomplete predictor sets were assigned to the appropriate predictor-set scenario (e.g., if TiO_2_ was missing, the sample was modelled using set 2s).

### Computational Framework

STATISTICA 12 (StatSoft) was used for statistical investigation and MARSpline regression modelling.

We applied the Multivariate Adaptive Regression Splines (MARSpline) method. It is a non-parametric regression procedure that proceeds from the assumption that the connection is built from a set of coefficients and basic functions that are completely “subordinate” to the regression data^54^. In our previous work, we found that MARSpline is the best method for the EMPC task^36^, so we use MARSpline in this work.

The parameters of MARSpline are the following:

- maximum number of basis function, 21;
- degree of interactions, 2;
- penalty, 2;
- threshold, 0.0005;

Pruning (backward elimination) was applied to remove basis functions that did not sufficiently improve the fit. No additional hyperparameter tuning was performed on the test subset; the parameters were kept constant for all 108 models to ensure comparability.

#### 3D block modelling

Composite intervals of 3 m length were created by averaging the assay values within each interval, weighted by sample length. Variogram modeling was performed on the composite data using the Mineframe software (Mining Institute of the Kola Science Centre Russian Academy of Sciences, and Mineframe Laboratory Ltd., http://mineframe.ru). For each predicted variable, experimental variograms were computed in the major directions of anisotropy. A spherical models with nugget, sill, and range were fitted manually. Ordinary kriging was used for interpolation with a maximum of 12 and minimum of 4 samples per block, and an elliptical search radius of 200 m in the strike direction, 150 m in the dip direction, and 50 m perpendicular to the ore zone. Cross-validation was used to assess the quality of variogram models (mean error close to 0, reduced squared error close to 1).

### Model validation

We split initial data for training and validation sub-sets randomly. We verified that the results were not overly sensitive by performing three alternative random splits for a subset of four key prediction models used in block modelling (nepheline, pyroxenes, “liebenerite”, SrO and REE_2_O_3_ in apatite), which yielded similar performance (standard deviation of R^2^ < 0.02).

Splitting proportion of initial datasets and size of total, training and validation subsets are provided in Table 3. For smaller datasets (e.g., magnetite chemistry, *n* = 61), a larger validation proportion (50%) was used to obtain a sufficiently sized validation set for reliable error estimation. We also clarify that the validation subset was used only for final evaluation; hyperparameters were fixed based on our prior work, so no separate test set was required. The different split proportions for different datasets (Table 4) are due to the need to maintain a sufficient number of validation samples for reliable error metrics, especially for smaller datasets (e.g., *n* = 61 for magnetite chemistry).

**Table 4:** Splitting proportion of initial datasets and size of total, training and validation subsets.

	Train/validation subsets	Total dataset	Train subset	Validation subset
Mineral composition	80/20	824	659	165
Apatite chemistry	80/20	237–241	189–193	44–48
Nepheline chemistry	70/30	84–91	77–64	20–27
Titanite chemistry	65/35	79	51	28
Magnetite chemistry	50/50	61	31	30

Prediction quality was assessed using correlation coefficients between predicted and measured values at a significance level of *p* < 0.05, following Russian Federation geological reporting standards (Tables 2 and 3)^55^. In Table 5, other regression error metrics (R^2^, MAE and RMSE) for the predictor set 1s are presented.

**Table 5:** Summary of prediction performance for the best predictor set (1s: P_2_O_5_, Al_2_O_3tot_, Al_2_O_3a.s_., TiO_2_).

Target	*R* ^2^	RMSE	MAE
*Mineral content (wt%)*			
Nepheline	0.98	3.57	2.25
Pyroxenes	0.85	3.57	2.61
Titanite	0.96	2.17	1.45
Titanomagnetite	0.72	6.61	1.80
Potassic feldspar	0.72	4.33	2.97
Ilmenite	0.20	0.56	0.36
Mica	0.15	1.40	0.82
Amphiboles	0.10	0.23	0.14
Lamprophyllite	0.12	0.15	0.09
«Liebenerite»	0.62	0.97	0.62
*Apatite chemistry (wt%)*			
SrO	0.76	0.60	0.49
REE_2_O_3_	0.58	0.10	0.08
F	0.14	0.13	0.11
*Nepheline chemistry (wt%)*			
Ga_2_O_3_	0.66	0.0007	0.0006
Rb_2_O	0.61	0.0036	0.0031
*Titanite chemistry (wt%)*			
Nb_2_O_5_	0.40	0.020	0.017
*Magnetite chemistry (wt%)*			
Fe_total_	0.58	0.85	0.70

RMSE is Root Mean Squared Error, MAE is Mean Absolute Error.

**Fig. 6:**
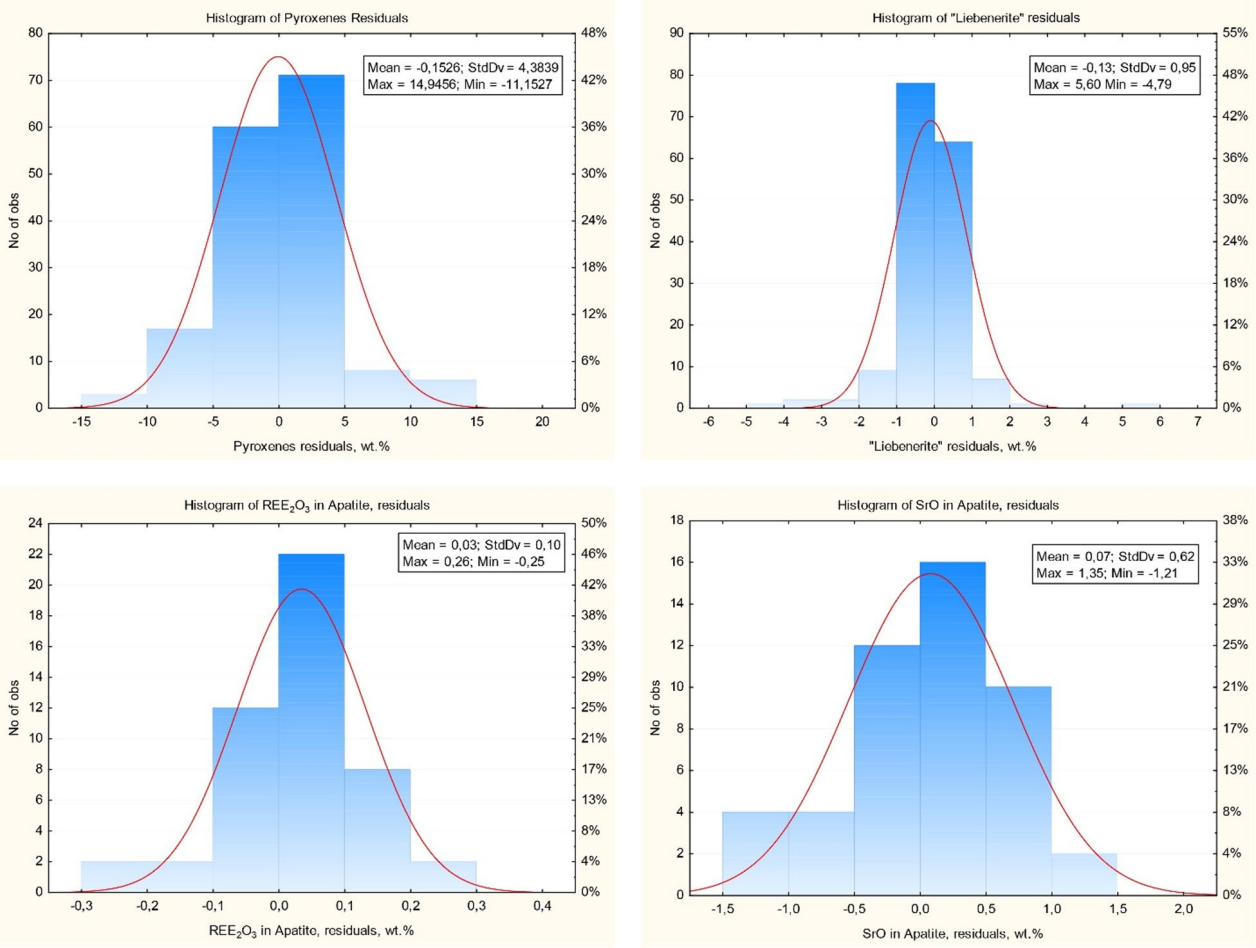
Histograms of residuals for key parameters (for the best predictor set 1s), validation subset.

To assess the distribution of prediction errors, we examined residuals (observed – predicted) for key target variables used in block modelling (nepheline, pyroxenes, “liebenerite”, SrO and REE_2_O_3_ in apatite). The residuals were approximately normally distributed with mean near zero, indicating no systematic bias. Histograms of residuals are provided in Fig. 6, and a summary of residual statistics is provided in Table 6. The variance of residuals was stable across the range of predicted values, with no pronounced heteroscedasticity.

**Table 6:** Summary of residual statistics for key parameters (for the best predictor set 1s), validation subset.

Pyroxenes	Mean	Median	Min	Max	StdDev	Skewness
	-0.15	0.2	-11.15	14.95	4.38	0.40
“Liebenerite”	-0.13	-0.12	-4.79	5.60	0.95	0.22
SrO in apatite	0.07	0.11	-1.21	1.35	0.62	-0.23
REE_2_O_3_ in apatite	0.03	0.04	-0.25	0.26	0.10	-0.47

Additional validation was performed using an independent set of apatite-nepheline ore samples analyzed by quantitative powder X-ray diffraction (pXRD).

Readers should note that the reported performance metrics are valid only for the compositional ranges and analytical methods represented in the training data (see Table 1). Extrapolation beyond these ranges or application to different ore domains should be done with caution and ideally validated with independent data.

## Supplementary Information

Below is the link to the electronic supplementary material.

Supplementary Material 1

## Data Availability

The datasets analysed during the current study are available from the corresponding author on reasonable request.

## References

[1] Ortiz, J. M. et al. Uncertainty and Value: Optimising Geometallurgical Performance Along the Mining Value Chain. *Elements* **19**, 377–383 (2023).

[2] Koch, P. H. & Rosenkranz, J. Sequential decision-making in mining and processing based on geometallurgical inputs. *Min. Eng.* **149**, 106262 (2020).

[3] Hunt, J., Berry, R., Becker, M. & Baumgartner, R. A Special Issue Dedicated to Geometallurgy: Preface. *Econ. Geol.* **114**, 1473–1479 (2019).

[4] Dominy, S. C., O’Connor, L., Parbhakar-Fox, A., Glass, H. J. & Purevgerel, S. Geometallurgy—A Route to More Resilient Mine Operations. *Minerals* **8**, 560 (2018).

[5] Frenzel, M., Baumgartner, R., Tolosana-Delgado, R., & Gutzmer, J. Geometallurgy: Present and Future.* Elements* **19**, 345–351 (2023).

[6] Lamberg, P. et al. Brisbane, Australia,. Building a geometallurgical model in iron ores using a mineralogical approach with liberation data. in *Proceedings of the Second AusIMM International Geometallurgy Conference* 317–324 (2013).

[7] Lund, C. & Lamberg, P. Geometallurgy – A tool for better resource efficiency. *Eur. Geologist*. **37**, 39–43 (2014).

[8] Lund, C., Lamberg, P. & Lindberg, T. Development of a geometallurgical framework to quantify mineral textures for process prediction. *Min. Eng.* **82**, 61–77 (2015).

[9] Bennett, D. W. & Munro, P. D. Perth, Australia,. Practical geometallurgy – and let there be light. in *Mill Operators Conference* (2024).

[10] Hoal, K. O. Getting the Geo into Geomet. *SEG Newsl.* **73**, 1, 11–15 (2008).

[11] Mena Silva, C. A., Ellefmo, S. L., Sandøy, R., Sørensen, B. E. & Aasly, K. A neural network approach for spatial variation assessment – A nepheline syenite case study. *Min. Eng.* **149**, 106178 (2020).

[12] Mishulovich, P. M. & Petrov, S. V. Geometallurgical models creation principles (in Russian with English abstract). *Vestn Saint Petersbg Univ. Earth Sci.* **64**, 249–266 (2019).

[13] Ellefmo, S. L., Aasly, K., Lang, A., Vezhapparambu, V. S. & Silva, C. A. M. Geometallurgical Concepts Used in Industrial Mineral Production. *Econ. Geol.* **114**, 1543–1554 (2019).

[14] Kalashnikov, A. O., Manukovskaya, D. V. & Stepenshchikov, D. G. How to Choose the Best Geometallurgical Strategy for Spatial Modeling of a Mineral Deposit. *Mining* **6**, 18 (2026).

[15] Lishchuk, V., Lamberg, P. & Lund, C. Classification of geometallurgical programs based on approach and purpose. in *13th Society for Geology Applied to Mineral Deposits (SGA) Biennial Meeting* 256–263 Society for Geology Applied to Mineral Deposits, Nancy, (2015).

[16] Lishchuk, V. *Geometallurgical Programs – Critical Evaluation of Applied Methods and Techniques* (Luleå University of Technology, 2016).

[17] Kots, G. A., Chernopyatov, S. F. & Shmanenkov, I. V. *Technological Sampling and Mapping of Mineral Deposits (in Russian)* (Nedra, 1980).

[18] Izoitko, V. M. *Technological Mineralogy and Ore Evaluation* (Nauka, 1997).

[19] Bachmann, K. Predictive geometallurgical modelling. *Technischen Universität Bergakademie Freiberg*. 10.13140/RG.2.2.13740.49288 (2020).

[20] Deutsch, J. L., Palmer, K., Deutsch, C. V., Szymanski, J. & Etsell, T. H. Spatial Modeling of Geometallurgical Properties: Techniques and a Case Study. *Nat. Resour. Res.* **25**, 161–181 (2016).

[21] Both, C. & Dimitrakopoulos, R. Applied Machine Learning for Geometallurgical Throughput Prediction — A Case Study Using Production Data at the Tropicana Gold Mining Complex. *Minerals* **11**, 1257 (2021).

[22] Bhuiyan, M., Esmaeili, K. & Ordóñez-Calderón, J. C. Evaluation of rock characterization tests as geometallurgical predictors of bond work index at the Tasiast Mine, Mauritania. *Min. Eng.* **175**, 107293 (2022).

[23] Lishchuk, V., Lund, C. & Ghorbani, Y. Evaluation and comparison of different machine-learning methods to integrate sparse process data into a spatial model in geometallurgy. *Min. Eng.* **134**, 156–165 (2019).

[24] Rajabinasab, B. & Asghari, O. Geometallurgical Domaining by Cluster Analysis: Iron Ore Deposit Case Study. *Nat. Resour. Res.* **28**, 665–684 (2019).

[25] Taboada, J., Matías, J. M., Ordóñez, C. & García, P. J. Creating a quality map of a slate deposit using support vector machines. *J. Comput. Appl. Math.* **204**, 84–94 (2007).

[26] Dehaine, Q., Filippov, L. O., Glass, H. J. & Rollinson, G. Rare-metal granites as a potential source of critical metals: A geometallurgical case study. *Ore Geol. Rev.* **104**, 384–402 (2019).

[27] Frenzel, M. et al. The geometallurgical assessment of by-products—geochemical proxies for the complex mineralogical deportment of indium at Neves-Corvo, Portugal. *Min. Depos.* **54**, 959–982 (2019).

[28] Whiten, B. Calculation of mineral composition from chemical assays. *Min. Process. Extr. Metall. Rev.* **29**, 83–97 (2007).

[29] Lund, C., Lamberg, P. & Lindberg, T. Practical way to quantify minerals from chemical assays at Malmberget iron ore operations – An important tool for the geometallurgical program. *Min. Eng.* **49**, 7–16 (2013).

[30] Ntlhabane, S. et al. Towards the development of an integrated modelling framework underpinned by mineralogy. *Min. Eng.* **116**, 123–131 (2018).

[31] Parian, M., Lamberg, P., Möckel, R. & Rosenkranz, J. Analysis of mineral grades for geometallurgy: Combined element-to-mineral conversion and quantitative X-ray diffraction. *Min. Eng.* **82**, 25–35 (2015).

[32] Minz, F., Bolin, N. J., Lamberg, P. & Wanhainen, C. Detailed characterisation of antimony mineralogy in a geometallurgical context at the Rockliden ore deposit, North-Central Sweden. *Min. Eng.* **52**, 95–103 (2013).

[33] Holmgren, D. C. & Marti, G. M. Applied microscopy and metallurgical forecasting at Los Bronces mine, Chile. in *2nd International Congress on Applied Mineralogy in the Mineral Industry* 407–419 Los Angeles, (1984).

[34] Navarra, A., Grammatikopoulos, T. & Waters, K. Incorporation of geometallurgical modelling into long-term production planning. *Min. Eng.* **120**, 118–126 (2018).

[35] Butcher, A. R., Dehaine, Q., Menzies, A. H. & Michaux, S. P. Characterisation of Ore Properties for Geometallurgy. *Elements* **19**, 352–358 (2023).

[36] Kalashnikov, A. O., Pakhomovsky, Y. A., Bazai, A. V., Mikhailova, J. A. & Konopleva, N. G. Rock-chemistry-to-mineral-properties conversion: machine learning approach. *Ore Geol. Rev.* **136**, 104292 (2021).

[37] Goryainov, P. M., Ivanyuk, G. Y. & Yakovenchuk, V. N. Tectonic percolation zones in the Khibiny massif: Morphology, geochemistry, and genesis. *Izv. - Phys. Solid Earth*. **34**, 822–827 (1998).

[38] Elbendary, A., Aleksandrova, T. & Nikolaeva, N. Influence of operating parameters on the flotation of the Khibiny Apatite-Nepheline Deposits. *J. Mater. Res. Technol.* **8**, 5080–5090 (2019).

[39] Elbendary, A. M., Aleksandrova, T. N. & Nikolaeva, N. V. Optimizing reagent regime in apatite-nepheline ore processing. *Min. Informational Anal. Bull.* 123–132. 10.25018/0236-1493-2020-10-0-123-132 (2020).

[40] Ivashchenkova, O. V. et al. The influence of the mineralogical properties on processing of apatite-nepheline ores. *Proc. Fersman Sci. Sess.* **20**, 570–578 (2023).

[41] Ivanov, S. M. *Optimization of exploration and calculation of reserves of complex apatite-nepheline deposits (in Russian)* (Moscow State University, 1987).

[42] Perekrest, I. I., Lazareva, L. F. & Ivanov, S. N. *Report on Detailed Exploration of Deep Horizons of the Koashva Deposit with a Radical Comprehensive Revaluation of Reserves of Apatite-Nepheline Ores as of 01.07.1985 (in Russian)*. (1985).

[43] Pleshakov, Y. V., Alekseyev, A. I. & Brylyakov, Y. Y. Nikolaev A. I. A combined processing technology for apatite-nepheline ores (in Russian with English abstract). *Obogashchenie Rud*. **46**, 15–17 (2004).

[44] Berman, I. I., Ivanov, S. N. & Kamenev, Е. А. *Geological Notes on the Methodology for Assessing the Contents of Accompanying Components and Minerals in Apatite-Nepheline Ores of the Rasvumchorr Plateau and Apatite Circus Deposits (in Russian)*. (1982).

[45] Ivanyuk, G. Y. et al. *Self-Organization of Ore Complexes (in Russian)* (Geokart-Geos, 2009).

[46] Snyatkova, O. L. et al. Report on the Results of a Geological Study and Geochemical Exploration for Rare Metals and Apatite on the Scale 1:50000, Carried out within the Khibiny Massif and Its Surrounding Area during 1979–1983 (in Russian). (1983).

[47] *State Report on the State and Use of Mineral Resources of the Russian Federation in 2018 (in Russian)*. (2019). http://www.mnr.gov.ru/upload/iblock/6e9/Государственныйдоклад-2018.pdf

[48] USGS. *Mineral Commodity Summaries 2022*. https://pubs.usgs.gov/periodicals/mcs2022/mcs2022.pdf (2022). 10.5066/P9KKMCP4

[49] Kamenev, E. A. *Prospecting, Exploration and Geological-Industrial Evaluation of Apatite Deposits of the Khibiny Type (in Russian)* (Nedra, 1987).

[50] Kamenev, Е.А. & Mineev, D.A. (Eds.)* New Khibiny Apatite Deposits (in Russian)*. (Nedra, Moscow, 1982).

[51] Konopleva, N. G. *Geology of the Koashva apatite-nepheline deposit (the Khibiny massif) (in Russian)* (Moscow State University, 2009).

[52] Goryainov, P. M., Konopleva, N. G., Ivanyuk, G. Y. & Yakovenchuk, V. N. Structural organization of the ore zone of the Koashva apatite-nepheline deposit (in Russian). *Otechestvennaya Geol.* **75**, 55–60 (2007).

[53] Gerasimova, L. et al. Titanite Ores of the Khibiny Apatite-Nepheline-Deposits: Selective Mining, Processing and Application for Titanosilicate Synthesis. *Minerals* **8**, 446 (2018).

[54] Friedman, J. H. Multivariate Adaptive Regression Splines. *Ann. Stat.* **19**, 1–67 (1991).

[55] Varlamov, A.I. (Ed.) *Guidelines for the Complex Study of Deposits and the Calculation of Reserves of By-Product Minerals and Components (in Russian)*. 15 (Moscow, Russia, 2007).

